# Modulating CT Attenuation of Polyvinyl Alcohol Cryogels for Individualized Training Phantoms in Interventional Radiology: A Proof-of-Concept Study

**DOI:** 10.3390/gels11080664

**Published:** 2025-08-20

**Authors:** Martin Volk, Ivan Vogt, Marilena Georgiades, Johanna Menhorn, Mathias Becker, Georg Rose, Maciej Pech, Oliver S. Grosser

**Affiliations:** 1 Department of Radiology and Nuclear Medicine, University Hospital Magdeburg and Medical Faculty of Otto-von-Guericke University, 39120 Magdeburg, Germany; 2 Research Campus STIMULATE, Otto-von-Guericke University, 39106 Magdeburg, Germany; 3 Chair in Healthcare Telematics and Medical Engineering, Institute of Medical Engineering, Faculty of Electrical Engineering and Information Technology, Otto-von-Guericke University, 39106 Magdeburg, Germany

**Keywords:** polyvinyl alcohol, PVA-C, phantom, CT, contrast agents, interventional, radiology, tissue-mimicking phantom

## Abstract

Anthropomorphic CT phantoms are essential training tools for interventional radiology. Given the high technical demands and stringent safety requirements in this field, realistic CT phantoms are vital simulation tools that support effective hands-on training, procedural planning, and risk mitigation. However, commercially available phantom geometries are limited in their scope. This study investigates the use of polyvinyl alcohol (PVA) to fabricate customizable training phantoms. PVA, a non-toxic material, can be processed into PVA cryogels (PVA-C) with tissue-like mechanical properties. We modified PVA-C (10 wt.% PVA) by incorporating various additives to adjust X-ray attenuation and achieve Hounsfield units (HUs) similar to different soft tissues. HU values were measured at X-ray tube voltages of 70, 120, and 150 kV. The inclusion of barium sulfate (e.g., *U* = 120 kV; 0.1–2 wt.%: 33.29 ± 5.45–323.72 ± 12.64 HU) and iohexol (e.g., *U* = 120 kV; 0.1–2 wt.%: 26.05 ± 4.74–161.99 ± 5.69 HU) significantly increased HU values. Iohexol produced more homogeneous HU distributions than barium sulfate and cellulose derivatives, with the latter having air gaps and inconsistencies. The tested formulations encompassed a wide range of soft tissue densities, with HU values varying significantly across the energy range (*p* < 0.001). While cellulose derivatives showed variable HU modulation, their primary role appears to be in modifying phantom texture and morphology rather than precise attenuation control. In conclusion, PVA-C demonstrates strong potential for use in interventional radiology training phantoms. Further studies may enhance phantom realism by replicating tissue textures, for example, through the incorporation of cellulose-based substances.

## 1. Introduction

Interventional radiology is a rapidly advancing medical discipline, characterized by image-guided, minimally invasive procedures [1]. Within this context, manual dexterity and proficiency in the use of fluoroscopic techniques for image guidance are essential parts of training for young professionals. Historically, training in interventional settings has involved the use of cadavers, animals, or supervised practice with authentic clinical cases [2]. Given the corresponding limitations (e.g., risk to patients when trainees learn basic craft in the clinical setting and time constraints imposed by clinical workflow), training has been identified as a key area for optimization [2,3,4]. One area of advanced training is the optimization of dose management in interventional procedures, with the aim of minimizing exposure levels for both patients and interventional radiologists [5,6]. In this context, modern technologies such as virtual and augmented reality are employed to enhance the quality of training and offset training limitations [2,7].

Nevertheless, hands-on practice with real phantoms remains a pivotal component in simulating interventional scenarios. However, the number of distinct commercially available biopsy phantom models is limited, and these models typically represent only simple geometric or anatomical structures [8,9]. As a result, it is not possible to adequately model a wide range of clinical cases (indications) or levels of complexity (e.g., defined by anatomical configurations). Consequently, the variability of training scenarios is severely constrained.

The use of modern manufacturing methods (e.g., rapid prototyping and 3D printing) to produce patient-specific training phantoms is one of the main approaches to overcoming this limitation. The optimization of these phantoms requires consideration of material-specific characteristics, in addition to mechanical and structural properties (e.g., the ability to provide adequate haptic feedback or properties relevant to ultrasound imaging). Materials used in interventional procedures involving CT-assisted navigation must also exhibit radiological equivalence to tissue (e.g., quantified by Hounsfield units (HUs)) to reproduce typical fluoroscopic image quality, including contrast between different anatomic structures.

A potential material under investigation is polyvinyl alcohol (PVA). Owing to its unique combination of properties, PVA has been the subject of extensive research, revealing a broad range of potential applications and leading to its widespread use in various industries, including food processing, water treatment, and building materials [10,11,12]. One notable application is the use of PVA-based cryogels (PVA-C) as phantom materials, applicable in MRI, ultrasound, and CT imaging [13,14,15,16,17,18,19]. As McGarry et al. have comprehensively reviewed, PVA-based cryogels have already been used as puncture and intervention phantoms in previous studies, demonstrating their suitability for simulating tissue characteristics relevant to interventional procedures [20]. Compared to commercial training phantoms, PVA-C can be produced at a fraction of the cost, which greatly facilitates the development of versatile, customized models for a wide range of interventional scenarios [9,21].

The aim of this study was to investigate the suitability of different contrast agents, applied at varying mass fractions, for their potential to modulate reconstructed HU values. To our knowledge, these specific combinations of substances have not yet been fully examined or optimized for X-ray-based imaging applications.

While different processing methods of PVA cryogels have been explored in MRI and CT phantoms [16,17], this study specifically focuses on the tailored modification of PVA-C samples for CT-guided interventions. The focus was on achieving soft-tissue-equivalent density values to support the visualization of clinically relevant contrast ratios. Additionally, the energy dependence of HU values was analyzed by performing measurements at different X-ray tube voltages.

## 2. Results and Discussion

Following dissolution and freeze–thaw processing, the base PVA-C material exhibited a milky, turbid appearance. The mean HU values for microwave-synthesized PVA-C were 22.93 ± 5.04 HU at *U* = 70 kV, 23.47 ± 5.71 HU at *U* = 120 kV, and 23.69 ± 4.91 HU at *U* = 150 kV. Post hoc analysis revealed significant differences between 70 kV and 120 kV (*p* = 0.0124) and between 70 kV and 150 kV (*p* < 0.001). No significant difference was observed between 120 kV and 150 kV (*p* = 0.2797).

### 2.1. HU Modification by Positive Contrast Agents

**Figure 1:**
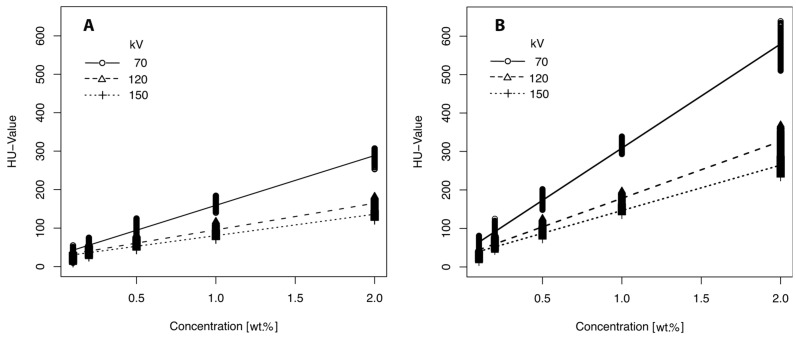
HU values of PVA-C samples showing the effect of different mass fractions (0.1–2 wt.%) and X-ray tube voltage. (**A**) Values of samples with iohexol and (**B**) with barium sulfate.

HU values were not normally distributed within the groups defined by X-ray tube voltage and additive concentration (*p* < 0.001). Both contrast agent concentration and X-ray tube voltage had a significant effect on the HU values of the PVA-C samples (*p* < 0.001, all effects, results from the Kruskal–Wallis test). Increasing the mass fraction of contrast agents resulted in a significant increase in HU values for samples containing iohexol (e.g., *U* = 120 kV; 0.1–2 wt.%: 26.05 ± 4.74–161.99 ± 5.69 HU, Figure 1A) and barium sulfate (e.g., *U* = 120 kV; 0.1–2 wt.%: 33.29 ± 5.45–323.72 ± 12.64 HU, Figure 1B). The increase in HU values was statistically significant between each consecutive concentration step for both additives (*p* < 0.001). Energy-dependent effects were significant across all concentrations (*p* < 0.001) and were more pronounced at higher contrast agent concentrations (Figure 1A,B). The influence of contrast media concentration and X-ray tube voltage on HU values was further analyzed using a generalized linear model (GLM; results summarized in Table 1).

**Figure 2:**
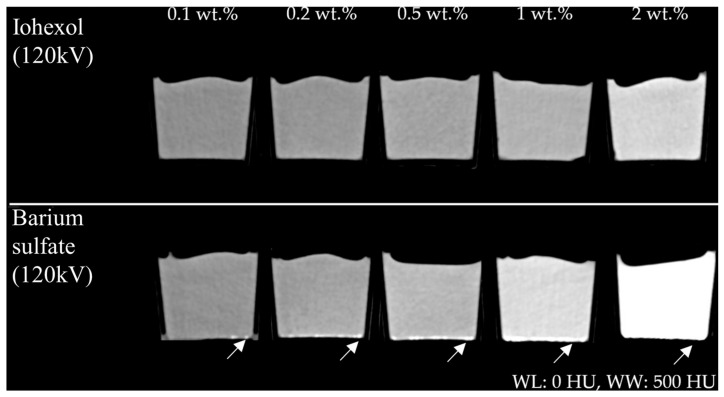
(**Upper row**) Reconstructed images of samples containing iohexol and (**lower row**) barium sulfate (each 0.1–2 wt.% at *U* = 120 kV, window level (WL): 0 HU, window width (WW): 500 HU). Note local dense deposits in the barium sulfate-containing probes (white arrow).

In the visual evaluation of the CT data, samples mixed with iohexol appeared to be very uniform. In general, barium sulfate also exhibited good homogeneity. However, samples containing barium sulfate showed denser deposits at the bottom of the container, most likely due to incomplete dispersion of barium sulfate (Figure 2). No subjective differences were noted between the samples regarding tactile or overall visual impression, except that barium sulfate imparted a distinctly white coloration to the PVA-C material.

The GLM analysis revealed that barium sulfate had a significantly stronger effect on HU values per unit concentration compared to iohexol, suggesting greater efficiency for applications requiring pronounced contrast enhancement. Both contrast agents showed a highly significant negative correlation (*p* < 0.001) with X-ray tube voltage, with barium sulfate exhibiting approximately twice the sensitivity to kV changes in tube voltage compared to iohexol. This difference in energy dependence indicates that the optimal phantom composition must be tailored to the selected contrast medium and intended application. For example, depending on the additive concentration, it is possible to simulate perfused organs after contrast administration or to contrast PVA-C-based bone-equivalent material.

In general, the relationship between positive contrast agents and PVA-C has not been extensively investigated in the literature, particularly concerning varying tube voltages. Iohexol, commonly used as a vascular contrast agent, is easier to process but more expensive than barium sulfate. Its extremely low solubility in water, non-toxic properties, and lack of systemic absorption make it suitable for use in imaging phantoms. However, certain limitations exist. The behavior of barium sulfate in PVA-based phantoms is more complex than simple dissolution. While a portion of the barium sulfate particles may remain suspended in the PVA–water solution, contributing to uniform turbidity, many particles tend to settle owing to their high density. This results in a partially heterogeneous distribution of the additive within the sample. To address this issue, various strategies can be employed to improve the overall uniformity of the sample, such as using finer particle sizes of barium sulfate to improve the suspension, using a modified stirring method to improve dispersion, and incorporating stabilizers such as cellulose derivatives [22]. Such approaches may lead to a more stable suspension of barium sulfate particles within the PVA matrix, resulting in more homogeneous contrast distribution throughout the PVA-C material. Further systematic studies evaluating these techniques are required to optimize the distribution of barium sulfate and ensure maximum homogeneity in future PVA-C phantom formulations.

### 2.2. HU Modification by Cellulosic Substances

**Figure 3:**
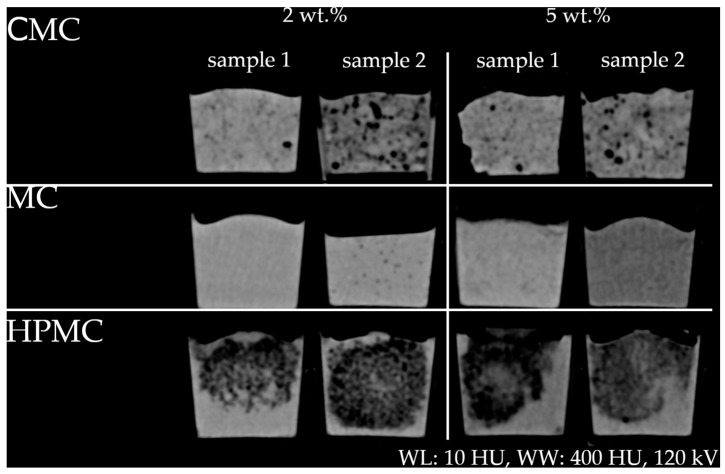
CT images of samples containing CMC, MC, and HPMC (2 wt.% and 5 wt.%) with different window settings (*U* = 120 kV). Typical broad window (WL: 10 HU, WW: 400 HU) for abdominal tissue.

For the cellulose derivatives examined, namely carboxymethyl cellulose (CMC), hydroxypropyl methylcellulose (HPMC), and methyl cellulose (MC), a reduction in HU values was observed. Owing to solubility limitations, samples from two independent batches of each cellulosic additive were analyzed to obtain an initial understanding of the resulting texture patterns. These batches exhibited altered pore architectures and increased variability in HU distribution (see Figure 3).

Unlike the results observed with positive contrast agents, no consistent trend in HU values was identified for CMC, HPMC, and MC with increasing mass fractions after the completion of the freeze–thaw cycles. A GLM analysis was not performed on the cellulose derivative samples because they did not show a clear linear or monotonic trend. Instead, they showed heterogeneous and non-linear behavior. Descriptive and qualitative assessments were therefore used to characterize their effects (see also Appendix A).

For example, CMC samples at 2 wt.% showed considerable variability, with HU values ranging from +10.03 HU (sample 1, *U* = 70 kV) to −58.57 HU (sample 2, *U* = 120 kV). At 5 wt.%, values ranged from −8.60 HU (sample 1, *U* = 150 kV) to −36.47 HU (sample 2, *U* = 150 kV), indicating poor reproducibility. MC samples at 2 wt.% exhibited more consistent, positive HU values (e.g., 29.79 HU and 21.23 HU at *U* = 70 kV), but at 5 wt.%, the values ranged from strongly positive (19.94 HU for sample 1, *U* = 70 kV) to strongly negative (−49.22 HU for sample 2, *U* = 70 kV). HPMC samples consistently showed negative HU values, ranging from −65.49 HU (sample 1, *U* = 70 kV) to −76.60 HU (sample 2, *U* = 150 kV) at 2 wt.% and from −82.23 HU (sample 2, *U* = 150 kV) to −98.06 HU (sample 1, *U* = 150 kV) at 5 wt.%. The high standard deviations (up to 63.11 HU for CMC sample 2, 5 wt.%, *U* = 150 kV, and 43.25 HU for HPMC sample 1, 2 wt.%, *U* = 120 kV) and interquartile ranges (IQRs) (up to 60 HU for CMC sample 2, 2 wt.%, *U* = 70 kV, and 56 HU for HPMC sample 1, 2 wt.%, *U* = 120 kV), reflecting pronounced inhomogeneity and the presence of large pore-like, air-filled structures. In contrast, MC samples showed much lower standard deviations (as low as 3.90 HU for sample 1, 2 wt.%, *U* = 150 kV) and IQRs (as low as 5 HU for sample 1, 2 wt.%, *U* = 150 kV), indicating a more homogeneous structure.

The observed variability and lack of consistent concentration effects, however, limit their use for precise HU adjustment. With increasing mass fractions of each cellulose derivative, cryogels became significantly harder. Additionally, higher mass fractions lead to poorer solubility of MC, HPMC, and especially CMC, which was evident during sample preparation. Despite these variations, all samples exhibited comparable structures characteristic of the respective cellulose derivative. The resulting probe texture imaged by CT was rated by experienced interventional radiologists. In MC samples (Figure 3), both readers reported a texture comparable to that typically observed in parenchymal organs (e.g., liver, spleen, and pancreas), depending on the specific contrast media phase and primary disease (e.g., fatty liver degeneration). For other material combinations (cellulosic derivative and wt.%), the readers propose a set of potential tissues (see Appendix A, Table A2).

It was found that water-soluble cellulose derivatives are potential substances for reducing HU values. In the literature, several biomedical applications of PVA combined with cellulose derivatives have been reported, including use as superabsorbents [23], in wastewater management [24], and in tissue engineering [25]. However, research on phantom materials suitable for biopsy procedures (e.g., ultrasound phantoms) has so far focused on non-soluble cellulose [15]. It was initially hypothesized that incorporating cellulose into PVA cryogels would reduce HU values by promoting air entrapment. Contrary to this expectation, the addition of cellulose derivatives resulted in significant texture modification and, in some cases, large air pore formation, leading to variable changes in effective density and X-ray attenuation across different samples. Whereas the initial mixture was visually homogeneous, the final solidified samples showed multiple texture patterns after the freeze–thaw cycles. These results were not reproducible when the samples were replicated. HU values did not consistently correlate with the amount of additive, showing unexpected variations between batches. The primary objective was not to create different textures using cellulose but to modulate HU values. The research focused on the potential of cellulose derivatives to influence X-ray attenuation properties of phantom materials. It is hypothesized that the different chemical properties of the cellulose derivatives, in combination with concentration, induced the formation of the observed texture patterns.

Although studies have examined the freeze–thaw behavior of cellulose (e.g., CMC) pertaining to intermolecular cross-linking [23,24], the interactions between cellulose derivatives and PVA under varying boundary conditions remain insufficiently understood. While cellulose derivatives exhibit complex and variable effects on HU values, their principal contribution lies in modifying the microstructure and texture, enhancing realism in phantom design. Both PVA and cellulose derivatives contain abundant hydroxyl groups, which enable hydrogen bonding that varies depending on the specific derivative. These intermolecular interactions likely influence the crystallinity, cross-link density, and overall microstructure of the polymer network. We hypothesize that, together with the differing affinities of these additives for water, these interactions affect the polymer structure formed during freeze–thaw cycles. This results in variations in pore size and distribution, leading to increased heterogeneity, which corresponds to the observed differences in CT attenuation and texture among samples with varying cellulose content. Factors such as freezing protocols and material choices require further investigation to elucidate their influence on the resulting PVA-C structure and properties. The preparation of reproducible cellulose–PVA phantoms requires careful attention not only to the freezing process but also to the initial solution preparation. A key aspect is the complex interaction between cellulose derivatives and water, which can introduce errors if not properly managed. Therefore, the incorporation of additives such as cellulose derivatives into water must be carefully controlled.

CMC quickly forms viscous gels in water, even at low concentrations. This rapid hydration requires vigorous stirring during addition to prevent lump formation, as CMC can easily agglomerate upon contact with water. In contrast, HPMC and MC exhibit thermos-reversible gelation behavior. They dissolve readily in cold water but form gels when heated above specific temperatures (approximately *T* = 68 °C for HPMC and *T* = 42 °C for MC). For homogeneous solutions, HPMC and MC should be added to cold water first, as adding them directly to hot water leads to immediate surface gelation, which can trap undissolved particles and result in inhomogeneities [26].

Despite these challenges, the unexpected heterogeneity observed in the samples revealed properties that could be leveraged to simulate not only homogeneous tissue groups but also more complex biological structures, such as malignant or necrotic tissue. (Appendix A, Table A2). Although the texture variations observed in the phantom materials resemble certain biological tissues, this correlation still needs to be validated using objective methods and larger expert cohorts. Planned studies should include histopathological analysis and advanced imaging techniques to determine the structural basis of these textures and their clinical relevance.

If reproducibility can be achieved through standardized protocols, controlled structures combining cellulose and PVA-C could be systematically engineered. This approach could also be expanded by incorporating positive contrast agents, enabling the simulation of diverse diagnostic scenarios (e.g., lesions with varying attenuation profiles). Although the current inconsistencies complicate reproducibility, they highlight the need for deeper investigation of factors influencing PVA–cellulose interactions, such as freezing rates, additive distribution, and cross-linking dynamics. These findings suggest that refined methodologies could enable controlled production of phantoms with tunable heterogeneity, better replicating the complex textures of real tissues. Such advancements would enhance the applicability of phantoms for validating imaging-guided biopsy systems.

The potential for uniformly reducing HU values in phantom materials may be more effectively achieved through alternative approaches. Rather than relying on air entrapment, which has proven challenging to control consistently, the use of low-density solid additives offers a promising avenue for exploration. Specifically, materials such as paraffin or latex, which have lower densities than water, could serve as effective agents for modulating HU values more predictably. This strategy focuses on directly altering the material’s overall density rather than generating air pockets. Such an approach may allow for more precise and reproducible control over the X-ray attenuation properties of phantom materials. Further studies are needed to evaluate the compatibility of various low-density additives with PVA cryogels to determine optimal concentrations and mixing protocols and assess their effects on the mechanical and imaging properties of the resulting phantoms.

### 2.3. CT Calibration

An energy-dependent effect from CT calibration on HU values was observed. The HU value distribution estimated from the CT data of the water sample showed a significant difference across all X-ray tube voltages (*p* < 0.001; *U* = 70 kV: 6.99 ± 5.76 HU, *U* = 120 kV: 0.48 ± 4.76 HU, and *U* = 150 kV: −0.77 ± 4.28 HU).

### 2.4. Further Material Characterization and Analytical Methods

Further research using analytical techniques such as scanning electron microscopy (SEM), thermogravimetric analysis (TGA), Fourier transform infrared spectroscopy (FTIR), differential scanning calorimetry (DSC), and the Brunauer–Emmett–Teller (BET) method is necessary [27,28,29]. While our study primarily focused on the application-oriented characteristics of PVA cryogels, the observed variations in texture and X-ray attenuation strongly suggest complex physicochemical interactions between PVA and, in particular, cellulose derivatives. A comprehensive understanding of these mechanisms will require future studies employing SEM to visualize microstructural arrangements, FTIR to characterize bonding interactions, BET analysis to quantify specific surface area and pore volume, and DSC to investigate thermal transitions and assess the degree of crystallinity affecting the polymer network. TGA could provide insights into thermal stability. These methods can provide valuable insights into the properties and structure of the resulting cryogels, enabling further optimization and advancement in the development of these phantom materials for medical imaging applications. As the microscopic changes induced by various additives compared to pure PVA-C remain insufficiently understood, the application of these techniques is essential to elucidate their effects. These methods facilitate a deeper understanding of how different additives alter PVA-C cryogels, thereby supporting the development of more precise and application-specific phantom materials for medical imaging.

As phantom construction is primarily about mimicking clinical situations, the setting for which they are to be constructed must be clearly emphasized. Owing to the real-time control required in CT-guided interventions and the significantly higher exposure from repeated image acquisition, careful attention must be paid to the patient’s radiation dose. Among other measures, tube current reduction is used, which affects image quality. However, this reduced image quality is generally considered acceptable for positional control of instruments rather than for diagnostic purposes [30]. This aspect should be critically considered when constructing PVA phantoms. Minor variations in freeze–thaw cross-linking conditions may yield statistically significant differences in imaging comparisons presented in this paper, but they are not necessarily relevant for training applications, where higher noise levels and non-diagnostic image quality are acceptable. The primary goal of developing and utilizing anthropomorphic phantoms is to enhance workflow efficiency and patient safety through various training scenarios. This approach can contribute to more dose-efficient interventions based on accumulated experience. Experienced interventional radiologists typically require fewer images for guidance, whereas less experienced practitioners may rely on more. From a radiation protection perspective, a well-practiced and standardized workflow during interventions is particularly important.

## 3. Conclusions

Phantom-based training has become increasingly important in interventional radiology to improve handling skills and optimize exposure procedures for both patients and staff using X-ray-based imaging modalities. However, the rigid geometry of standard prefabricated models severely limits training options. This study investigated PVA-C, a material previously also used for phantoms in other imaging modalities (e.g., MRI and ultrasound), for its suitability in CT-guided procedures.

PVA is a versatile, non-toxic, and durable polymer that enables the fabrication of customized phantoms with adaptable geometries and compositions closely mimicking human tissue. This makes it possible to produce patient-specific phantoms for training medical professionals in various procedures or for preparation of complex cases. In this context, we examined the feasibility of modifying the attenuation properties of PVA-C to mimic human tissue density in CT imaging and validated the effects of kV and weight percent of additives on HU values. The examinations revealed a significant correlation between additive concentration and reconstructed HU values for each of the positive contrast media (iohexol and barium sulfate). Additionally, the modification of texture in CT images through the addition of cellulose derivatives was examined.

These findings confirm that by selecting suitable density-regulating additives, PVA cryogels can be tuned to emulate various human-like HU values and achieve either highly homogeneous (e.g., with iohexol) or intentionally heterogeneous (e.g., with barium sulfate or cellulose derivatives) structures. This flexibility is particularly advantageous for applications that require simulating inhomogeneous tissues, tumors, fibrosis, or other lesions. To achieve precise control of low HU values, alternative materials with lower effective density could be utilized instead of creating air-filled pores, or the manufacturing methodology itself may need to be modified. The specific parameters and number of additives that directly influence the properties of the material still require further investigation.

## 4. Materials and Methods

PVA is a semi-crystalline, thermoplastic, biodegradable, and non-toxic synthetic polymer. PVA-C is formed by dissolving PVA granules in water and inducing cross-linking through freeze–thaw cycles, resulting in a semi-opaque, stable, three-dimensional tissue-like network [31,32,33]. The PVA used in this study was Kuraray Poval^®^ 15-99 (Kuraray Europe GmbH, Frankfurt am Main, Germany), with a mean degree of hydrolysis of *h* = 99% (range: 99.0–99.8%) and a mean viscosity of η = 15 mPa·s, measured according to DIN 53189/JIS K 6726 standards in a 4 wt.% aqueous solution at *T* = 20 °C. 

### 4.1. General Processing of PVA Probes

PVA granules were dissolved in distilled water using a microwave oven (Samsung MC28M6055CK/EG, Suwon, South Korea). In this setup, PVA batches were heated at a power level of *P* = 300 W. This method has proved to be a reproducible alternative to the conventional hotplate dissolution method with manual stirring, as previously demonstrated for MRI phantoms [16]. Initial results have also shown its applicability for CT imaging [17].

**Figure 4:**
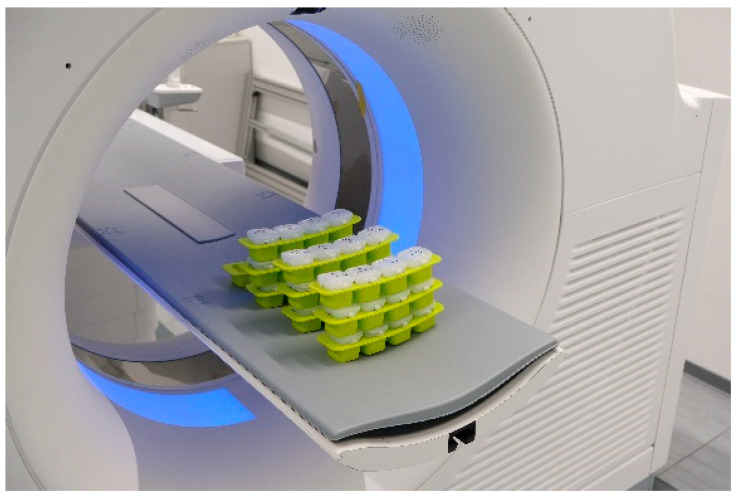
Sample container setup in the CT gantry.

Water loss during heating was minimized using a microwave-compatible lid with a steam vent; any evaporated water was replenished as needed. The resulting solutions were then transferred into freezable polypropylene (PP) containers (50 mm × 50 mm × 45 mm, *m* = 40 g, Rotho Kunststoff GmbH, Görwihl, Germany) (Figure 4). After cooling to room temperature, additives were incorporated into the PVA solution as required. Following molding, the samples were frozen at *T*_freeze_ = −18 °C for *t*_freeze_ = 12 h, followed by thawing at ambient temperature (*T*_thaw_ = 20 °C) for *t*_thaw_ = 12 h [34]. All samples were standardized to undergo two freeze–thaw cycles and a PVA concentration of 10 wt.%.

### 4.2. Modulation of X-Ray Attenuation

Individual PVA solutions were prepared in accordance with the fabrication procedure previously described, with the mass fractions maintained according to the desired concentration of the additive.

Modification of X-ray attenuation was achieved using barium sulfate (pch distribution, Blumberg, Germany) and iodine-based iohexol liquid (GE Accupaque 350, GE Healthcare Buchler GmbH & Co., KG, Braunschweig, Germany). Both agents are well-established contrast media in radiology [35,36,37,38]. Additionally, cellulose derivatives were incorporated to reduce the density of PVA-C and create a low-density, tissue-equivalent material (e.g., simulated lung or adipose tissue). We hypothesized that the addition of various cellulose derivatives would introduce microscopic air inclusions, thereby decreasing effective attenuation and enabling the simulation of characteristics typical of low-density biological tissues. In general, cellulose is particularly well suited for biomedical applications owing to its hydrophilic nature, biocompatibility, and ability to form stable, interconnected porous structures [39]. The molecular interactions between cellulose derivatives, such as CMC and HPMC, and PVA during freeze–thaw cycles play a key role in determining the pore architecture of the resulting cryogels. This process is influenced by mechanisms such as hydrogen bonding and ice templating, wherein ice crystals act as transient porogens. As the system undergoes freezing, cryo-concentration occurs, enhancing polymer interactions within the unfrozen microphases and leading to the development of distinct pore characteristics compared to cryogels composed solely of PVA. Some cellulose derivatives are capable of forming additional hydrogen bonds with PVA. For example, CMC [25] can form such bonds via its carboxyl groups, whereas MC [40] can do so through its methoxy groups. In contrast, HPMC cannot form compatible blends with PVA owing to its methoxy and hydroxypropyl substitution pattern, which precludes significant intermolecular hydrogen bonding [41]. These differences in molecular interactions further influence the structural and mechanical properties of cryogel networks.

In this study, food-grade HPMC (viscosity of η = 4000 mPa·s), MC (η = 75,000 mPa·s), and CMC (η = 5000–6000 mPa·s) were employed [42,43,44]. For complete dissolution, MC and HPMC were added slowly to the cooled PVA solution at room temperature (*T* = 20 °C), as both derivatives are readily soluble in cold water. This procedure prevents premature gelation and ensures full hydration, yielding a lump-free solution without additional heating or cooling. This method was chosen because both MC and HPMC dissolve more efficiently at lower temperatures, whereas elevated temperatures can induce gelation and hinder proper dissolution. CMC was similarly added to the cooled water–PVA mixture at room temperature, as carboxymethylcellulose is highly soluble in cold water and, unlike MC and HPMC, does not form gels upon heating.

All required mass fractions were measured using a precision scale (Kern EMB 2000-2, KERN & SOHN GmbH, Balingen-Frommern, Germany). Each sample containing an additive was mixed for 5 min using a hand mixer (Rosenstein & Söhne, Buggingen, Germany).

### 4.3. CT Imaging

CT imaging was performed using a clinical CT scanner (SOMATOM X.cite CT, Siemens Healthineers, Erlangen, Germany). The scanning protocol followed the manufacturer’s standard for abdominal imaging, with pitch = 0.8, rotation time *t*_rot_ = 0.5 s, primary collimation of 64 × 0.6 mm, and automatic exposure control (CAREDose4D, with manual kV setting). CT scans were acquired at different X-ray tube voltage settings (*U* = 70, 120, and 150 kV), whereas all other parameters were maintained constant. CT images were reconstructed using a matrix size of 512 × 696 voxels (pixel spacing = 0.373 × 0.373 mm) and a slice thickness of *z* = 5 mm. Reconstruction was performed using iterative image reconstruction (ADMIRE, level 3) with a soft tissue kernel (Br40d).

HU values were evaluated from the reconstructed CT images using MATLAB (version: 2024b, The MathWorks, Inc., Natick, MA, USA). Each sample was segmented using a semi-automatic algorithm based on intensity thresholding to separate the PVA-C material from the surrounding environment (e.g., air and sample container). To avoid artifacts, such as those caused by uneven surfaces, the three-dimensional HU value distribution was extracted from a volume of interest (VOI) of *V* = (1 × 1 × 1) cm^3^, positioned at the center of mass of each segmented PVA-C sample. Segmentation and VOI placement were verified through visual inspection. For each sample, *n* = 1458 values from the VOI were analyzed.

In addition, a water-filled reference container (identical PP container) was scanned at all analyzed X-ray tube voltages to account for potential HU calibration bias in the measured probe sample densities.

All reconstructed slices of the examined samples can be found in the Supplementary Files for more in-depth analysis.

### 4.4. Statistics

Statistical analysis was performed using R (version 4.4.1; R Foundation for Statistical Computing, Vienna, Austria). HU values were analyzed based on the combination of the PVA sample and the CT scan protocol. Descriptive statistics are presented as mean and standard deviation or as median, IQR, and range, as appropriate. Normality was assessed using the Kolmogorov–Smirnov test. For non-normally distributed data, the Kruskal–Wallis test was used to evaluate the effects of X-ray tube voltage, mass fraction variations, and additive type. Effects were further analyzed using a GLM for each additive.

The Bonferroni–Holm correction was applied after the pairwise *t*-test to adjust for multiple comparisons in case of statistical significance. All tests were two-sided, with a significance level set at α = 0.05. 

## Figures and Tables

**Table 1:** Results of GLM analysis describing the effects of contrast media concentration and X-ray tube voltage on the HU values of PVA-C samples for barium sulfate and iohexol.

Contrast Agent	Contrast Media Concentration	kV	Intercept	R^2^
**Barium sulfate**	179.16 (178.23–180.09) *p* < 0.001	−1.61 (from −1.63 to −1.59) *p* < 0.001	214.74 (212.33–217.15) *p* < 0.001	0.886
**Iohexol**	84.63 (84.17–85.09) *p* < 0.001	−0.78 (from −0.79 to −0.77) *p* < 0.001	115.84 (114.64–117.04) *p* < 0.001	0.876

All values are reported as estimates, along with their corresponding confidence intervals (CIs) and levels of significance. A total of 21,870 voxel values were analyzed per contrast agent using GLM.

## Data Availability

All CT datasets generated and analyzed during this study are available from the corresponding author upon reasonable request. Due to the large number of samples and the complexity of the dataset, detailed mapping of each sample to its specific material composition is necessary for meaningful interpretation. This information, including comprehensive explanations and sample assignments, will be provided upon request to ensure proper understanding and use of the data.

## Supplementary Materials

The following supporting information can be downloaded at https://www.mdpi.com/article/10.3390/gels11080664/s1. All the samples relevant to the manuscript have been extracted from the DICOM image data and included as supplementary .mat files. This supplementary file contains the complete sample datasets for all tested tube voltages, as referenced in the manuscript, and allows detailed analysis of material compositions.

## Appendix A

**Table A1:** Calculated mean, standard deviations, and IQR of the HU values from PVA-C samples with different masses of CMC, MC, and HPMC and tube voltages.

Concentration			2 wt.%		5 wt.%	
Cellulose	kV	Sample	Mean	IQR	Mean	IQR
**CMC**	70	1	10.03 ± 14.23	16	−9.22 ± 18.72	21
		2	−46.76 ± 48.68	60	−31.63 ± 58.46	41
	120	1	10.66 ± 13.85	15	−10.19 ± 17.43	20
		2	−58.57 ± 46.04	47	−30.68 ± 51.54	36
	150	1	10.57 ± 14.34	14	−8.60 ± 17.91	20
		2	−42.84 ± 47.02	56	−36.47 ± 63.11	43
**MC**	70	1	29.79 ± 6.54	8	19.94 ± 6.15	8
		2	21.23 ± 9.23	7	−49.22 ± 7.14	10
	120	1	28.43 ± 4.67	7	18.29 ± 6.24	8
		2	23.07 ± 9.33	7	−46.49 ± 6.09	8
	150	1	28.14 ± 3.90	5	18.08 ± 6.29	9
		2	23.04 ± 8.81	7	−47.88 ± 5.67	8
**HPMC**	70	1	−65.49 ± 38.76	54	−96.36 ± 34.89	54
		2	−74.64 ± 29.12	43	−84.98 ± 29.24	37
	120	1	−67.04 ± 43.25	56	−94.96 ± 26.75	37
		2	−74.56 ± 31.44	46	−86.86 ± 29.52	37
	150	1	−68.12 ± 39.82	51	−98.06 ± 30.50	43
		2	−76.60 ± 29.07	42	−82.23 ± 30.33	39

**Table A2:** Evaluation of the structural pattern produced with different cellulose–PVA samples in terms of similarity to biological tissue.

Concentration			2 wt.%	5 wt.%
Cellulose	Radiologist	Sample	Description	Description
**CMC**	1	1	Liver parenchyma in cirrhosis	Granular intestinal contents
		2	Granular intestinal contents	
	2	1	Fatty muscles	Fibrosis
		2	Corpostasis	Corpostasis
**MC**	1		**In general** Parenchymal organs: liver, spleen, pancreas; Depending on the KM phase and, for example, fatty degeneration (liver)	
		1	Tumor, cerebral cortex	Lobular parenchyma (pancreas)
		2	-	Steatosis hepatis or parenchyma without contrast medium
	2	1 2	Liver/spleen Cysts/metastases in the liver	Metastases in the liver, fatty liver
**HPMC**	1		**In general** Intestinal contents (due to granular consistency); Lung parenchyma (hypodense/black areas) with consolidation (e.g., pneumonia) or fibrosis (honeycomb lung, sample 2 [2.wt%]) in soft tissue window; Osteolysis/bone destruction in the bone window, e.g., bone tumor/metastasis.	
	2	1 2	Fatty muscles Honeycomb lung	- Osteolysis
